# Viral dew: Phase separation and the formation of viral replication compartments

**DOI:** 10.1371/journal.ppat.1011145

**Published:** 2023-02-16

**Authors:** Jens B. Bosse, Wolfram Brune

**Affiliations:** 1 Leibniz Institute of Virology (LIV), Hamburg, Germany; 2 Centre for Structural Systems Biology, Hamburg, Germany; 3 Institute of Virology, Hannover Medical School, Hannover, Germany; 4 Cluster of Excellence RESIST (EXC 2155), Hannover Medical School, Hannover, Germany; 5 German Center for Infection Research (DZIF), Partner Site Hamburg-Lübeck-Borstel-Riems, Germany; University of Arizona, UNITED STATES

## Introduction

Cellular life is based on compartmentalization to sequester biochemical reactions. Two kinds of cellular compartments exist: membrane-enclosed and membrane-less compartments. The cytoplasm of eukaryotic cells harbors many membrane-enclosed compartments, such as the endoplasmic reticulum, lysosomes, and peroxisomes. At the same time, the cytoplasm and especially the nucleus are compartmentalized by membrane-less structures, such as nucleoli, promyelocytic leukemia nuclear bodies (PML-NBs), nuclear speckles, and others. Not surprisingly, viruses hijack both compartment types to facilitate their replication. While the genesis of membrane-enclosed compartments has been studied for decades, research only recently focused on the biophysical properties of membrane-less compartments. Here, we outline the basic principles of their formation and how viruses generate such compartments for replication and morphogenesis. As excellent in-depth reviews have recently been published [1–3], this article is meant to serve as a brief introduction to the field.

## What is liquid–liquid phase separation?

Seminal work by Brangwynne and colleagues [4,5] showed that P granules, perinuclear membrane-less compartments in *C*. *elegans*, dissolved and condensed during embryonic development. They found that P granules show properties of fluids as they fuse and regain sphericity, drip, and can wet surfaces—typical properties of fluids exhibiting surface tension. Moreover, photobleaching experiments on the compartments demonstrated a fast fluorescence recovery, indicating the existence of an equilibrium between the soluble and the condensed phase. Importantly, P granules dissolved and reformed dependent on the local protein concentration, which is a classic sign of phase transitions [4]. Hence, the concept of liquid–liquid phase separation (LLPS) was introduced into cell biology. In its simplest form, phase separation (PS) describes the demixing of 2 components into 2 separate phases at a certain concentration threshold, dependent on factors such as temperature, pH, salt concentration, and crowding agents. In layman’s terms, PS occurs when molecules of component A “like” to interact with themselves more than with the surrounding component B. An intuitive example is dew formation: When humid air cools, air and water molecules separate and form 2 phases: a dense phase (dew) and a more dilute phase (vapor). Similarly, proteins can form biomolecular condensates as a concentrated phase that excludes much of the surrounding solvent. The conditions at which this separation happens depend on the properties of the molecule(s) involved. Many proteins can be driven into PS if concentrations are artificially increased beyond their saturation concentration [6]. The critical question is if this concentration reflects a physiological or pathological cellular state.

**Fig 1:**
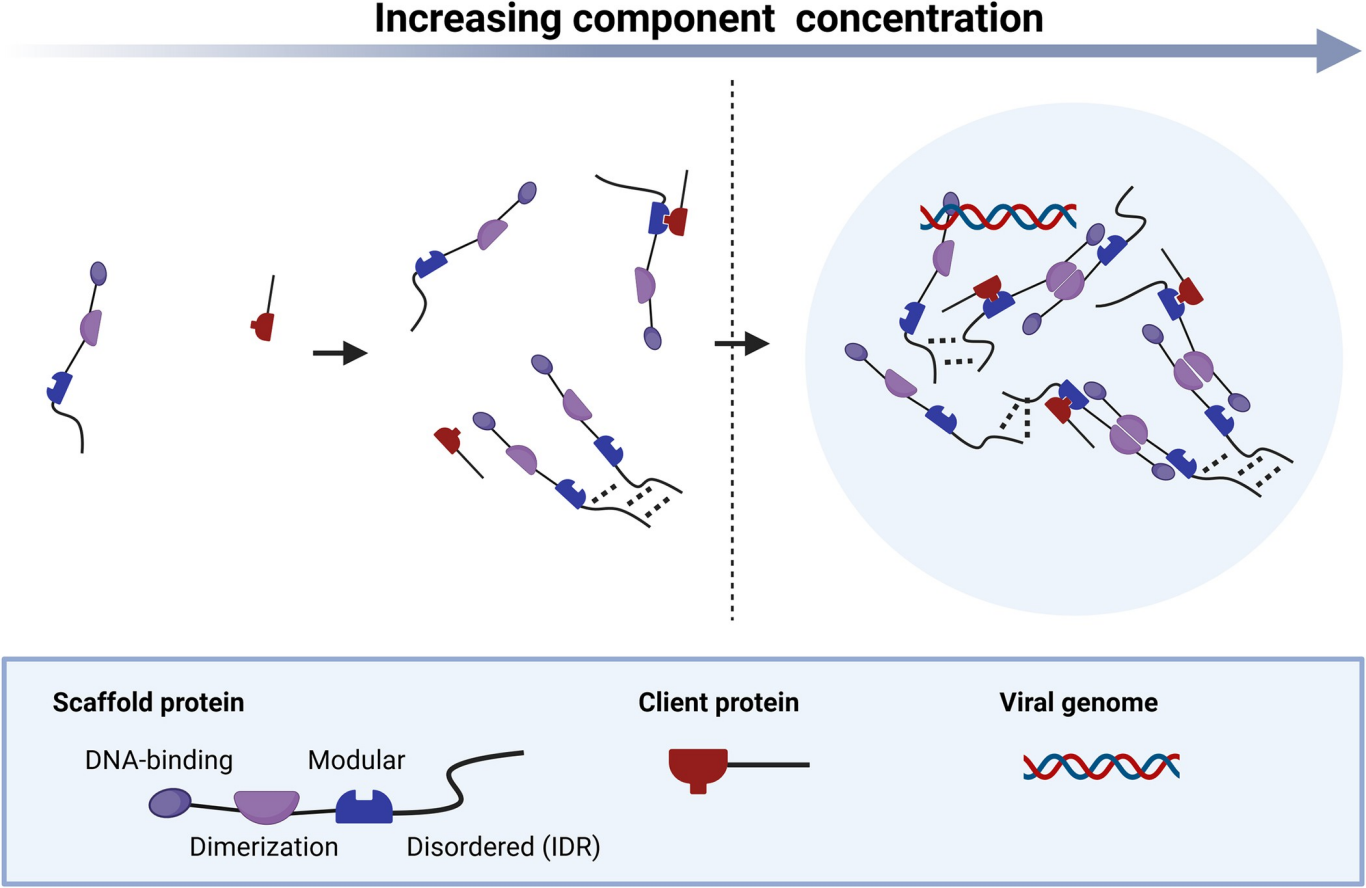
Interaction of multivalent molecules leading to LLPS. At constant environmental conditions (e.g., temperature, ionic strength, pH), the propensity of a multivalent scaffold protein to undergo LLPS only depends on its concentration. Crossing the protein’s specific concentration threshold (dashed line) leads to droplet formation resulting in a qualitative change upon a small, linear quantitative change. Created with Biorender.com. LLPS, liquid–liquid phase separation.

Multivalency of a protein is essential for PS (Fig 1). The degree of multivalency determines a protein’s PS behavior at physiological concentrations. Multivalency is mediated by stickers that are separated by spacers [7]. Stickers mediate interactions between molecules and can be located in structural domains as well as in intrinsically disordered regions (IDRs). IDRs are often important parts of phase-separating proteins as they contribute to multivalency by mediating weak interactions. However, not all IDRs mediate PS [8]. In contrast, spacers modulate the solubility of a molecule in a solvent. As valency (the number of stickers) increases, the general propensity for PS also increases. If the number of interactions crosses a saturation threshold, they overpower the interactions with the solvent resulting in PS [8]. Often, proteins undergoing PS contain different kinds of stickers, such as multimerization domains as well as certain combinations of residues within IDRs. Cellular polymers such as RNA and DNA can promote PS of proteins by crosslinking molecules and providing additional multivalency. Moreover, posttranslational modifications can modulate the interactive forces both positively (e.g., by adding stickers such as SUMO [9]) and negatively by masking stickers or increasing solubility (reviewed in [10]). Importantly, the cumulative quality and strength of the interactions determine the properties of the resulting interaction network. Multivalent proteins that drive PS are also called scaffolds, while proteins that partition into the condensed phase but do not contribute to PS are called clients.

Not all membrane-less compartments are fluid and formed by LLPS. Indeed, LLPS is a special case of condensate formation [11]. There are other forms of biomolecular condensates, such as gels and aggregates [11–13], that can form depending on the collective molecule’s properties, its concentration, and its environment. For example, percolation leads to networked gels if the critical concentration of a molecule for percolation is lower than the concentration threshold for phase separation (reviewed in [11]). Moreover, these transitions are dependent on further environmental factors such as temperature, ionic strength, and pH of the surrounding solvent. Finally, some liquid compartments can change their properties over time as outlined below.

## What is the function of LLPS in cell biology?

Cellular life depends on a steady state of dynamic processes. This is essential to react to cues rapidly. If a system is close to a phase transition threshold, small changes in the protein concentration or environment (changes in pH, salt concentration, temperature) are sufficient to induce condensation and the formation of novel compartments. Condensation leads to a qualitative change through minimal quantitative changes (binary response upon minimal analogous changes). The advantage for the cell becomes immediately apparent, as a slight change in the protein’s local concentration can induce compartment formation or dissolution. Many cellular processes depend on LLPS, such as transcription, RNA processing, DNA damage response, or intracellular signaling (reviewed in [3,13,14]).

## Do viruses make use of LLPS and what for?

Many phase-separated membrane-less compartments, such as P bodies, Cajal bodies, nucleoli, and nuclear speckles, contain proteins rich in IDRs and RNA and play a role in RNA synthesis and processing [12]. The same is true for replication compartments (RCs) of RNA viruses, which are sometimes called viral factories, virosomes, or inclusion bodies. Hence, it was reasonable to speculate that viral RCs could have properties of liquid organelles. This was first shown to be the case for Negri bodies, the characteristic inclusion bodies of rabies virus-infected cells [15]. Since then, several RNA viruses such as vesicular stomatitis virus, measles virus, rotavirus, influenza virus, and SARS coronavirus have been shown to form phase-separated compartments [16–22], and others are suspected to do the same (reviewed in [2,3]). PS is usually driven by the viral RNA-binding nucleoprotein, alone or in combination with other viral proteins. Besides serving as RCs, viral phase-separated compartments can also serve as sites for viral genome packaging or assembly.

More recent studies have shown that intranuclear RCs of certain DNA viruses also display properties of liquid organelles. Nascent adenovirus (AdV) RCs resemble spherical droplets that fuse to form larger RCs [23]. The viral DNA-binding protein, which contains an N-terminal IDR, can form similar structures when expressed in transfected cells, suggesting that it is responsible for the liquid properties of the viral RCs [23]. Similarly, the herpes simplex virus type 1 (HSV-1) ICP4 protein, a DNA-binding transcription factor with an IDR, forms droplets with liquid-like properties in the nucleus of transfected cells [24]. Another herpesvirus, human cytomegalovirus (HCMV), uses its UL112-113 proteins as a scaffold to form liquid RCs that are essential for viral replication [25]. The UL112-113 proteins exist in 4 isoforms with a common self-interacting domain at the N-terminus and extended IDRs, both of which are required for LLPS. The UL112-113 proteins are DNA-binding proteins [26], and viral DNA induces local clustering of these proteins, nucleating the formation of phase-separated droplets. The viral UL44 protein, a subunit of the viral DNA polymerase, accumulates as a client protein in these droplets through its own IDR, indicating that PS helps to recruit factors necessary for viral replication [25]. A recent study demonstrated that the human papillomavirus DNA-binding E2 protein forms biomolecular condensates together with the cellular p53 protein as a means to modulate viral transcription and replication [27].

Viruses might benefit in many ways from phase-separated RCs. Factors needed for viral gene transcription and genome replication could be concentrated, while detrimental factors, such as pattern recognition receptors, signaling molecules, and antiviral effector molecules could be excluded. However, RCs may also serve to sequester cellular antiviral factors, thereby preventing them from performing their physiological functions. In any case, RCs are likely to provide a protected space conducive to viral replication. For example, PML-NBs and histones associate with incoming viral DNA in the nucleus and suppress viral transcription [28]. Many DNA viruses counteract this by disrupting PML-NBs [29] and forming RCs. Moreover, the surface tension of phase-separated RCs provides the mechanical force to displace cellular chromatin. On the other hand, the formation of viral RCs creates new interfaces to the surrounding cytoplasm or nucleoplasm, which might be detected by cellular sensors. Whether such sensors exist and how they might discriminate between cellular and viral phase-separated compartments remains to be determined. It is also unknown whether all viral RCs arise de novo. Some may originate from preexisting phase-separated compartments. For example, the innate immune sensors cGAS and RIG-I recognize viral DNA and RNA, respectively, and accumulate in liquid condensates in the cytoplasm (reviewed in [30]). In the nucleus, viral DNA associates with PML-NBs, which have properties of liquid condensates [9].

While the potential benefits of phase-separated RCs are obvious and conceptually compelling, experimental evidence for these benefits remains sparse. One example is the HCMV UL44 protein that is enriched in RCs via its IDR [25]. On the other hand, how cellular antiviral proteins such as DAXX are specifically excluded from RCs [31] has yet to be understood. Once the proteomes of various viral RCs have been determined and their protein properties have been studied, we will better understand how PS contributes to protein inclusion or exclusion.

## Open questions

**Fig 2:**
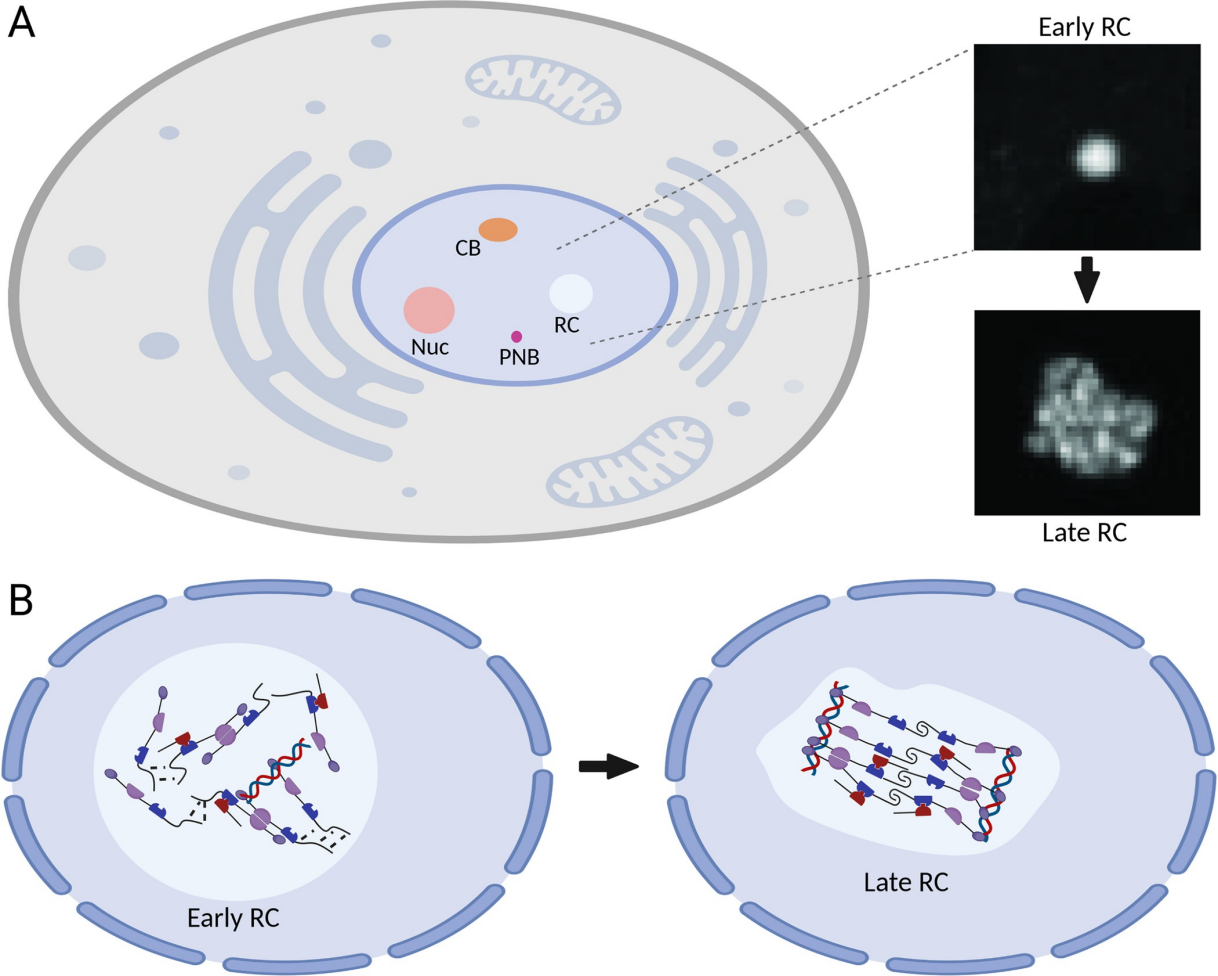
Maturation of viral replication compartments. (A) The nucleus contains membrane-less compartments such as Nuc, CBs, PML-NBs (PNB), and RCs of DNA viruses. In the case of HCMV [25], early RCs exhibit fluid properties consistent with being formed by LLPS. After the onset of viral DNA replication, these properties change, resulting in the hardening of late RCs. (B) Hypothetical model. In early RCs, weak and transient interactions predominate, resulting in fluid properties (left). In late RCs, the increased quantity of viral DNA, accumulation of client proteins, and posttranslational modifications of the scaffold proteins may result in tighter interactions of the molecules and the formation of more ordered molecular assemblies (right). Created with Biorender.com. CB, Cajal body; HCMV, human cytomegalovirus; LLPS, liquid–liquid phase separation; Nuc, nucleoli; PML-NB, promyelocytic leukemia nuclear body; RC, replication compartment.

The study of LLPS, or in a broader sense, the study of the polymer properties of cellular compounds, is still a young field with few well-established tools and imprecise terminology. Widely accepted standards have emerged only recently [32]. Many reports are descriptive, and therefore, future studies will need to focus on a better mechanistic understanding. Some critics have gone so far as to question the existence of phase-separated compartments in the complex environment of a living cell [33]. One important caveat is that scaffold proteins (PS drivers) were often identified in vitro using simplified model systems consisting of few components. In some cases, it remains unclear if these drivers play the proposed role in the more complex cellular environment [32]. The recent interest in PS in cell biology underlines the need to illuminate the physical underpinnings of membrane-less compartment formation. Moreover, proteins undergoing LLPS show a phenomenon called maturation or aging as time-dependent viscoelastic fluids [11] where they transition into solids or even amyloids [34]. Finally, changing composition will change the material properties of the condensed phases. Several studies reported that the liquid properties of viral RCs change over time. In rotavirus, the hyperphosphorylation of NSP5 leads to less fluid RCs at late times of infection [22]. The RCs of AdV and HCMV also become less fluid and more gel-like after the onset of viral DNA replication [23,25] (Fig 2). The reasons for these transitions have yet to be fully understood. However, it seems likely that viral gene transcription, genome amplification, and posttranslational modifications of the phase-separating viral proteins are involved. Therefore, it will be crucial to understand the complete composition of membrane-less compartments and develop tools to probe their properties within the cell. Viruses have played an instrumental role in uncovering many basic principles of cell biology. Although the importance of LLPS was first demonstrated with cellular membrane-less organelles, viral RCs could serve as excellent models for studying such compartments in living cells. Viral RCs form de novo upon viral infection and change their properties over time in defined ways as the infection progresses. Notably, the viral proteins that form viral RCs are more accessible to manipulation than the corresponding cellular proteins, making them ideal tools for dissecting the mechanisms and dynamics of membrane-less compartment formation. Understanding the basic principles of membrane-less compartment formation might also lead to novel antiviral treatment strategies as first results indicate [35,36].
